# Use of Sodium Butyrate and Its Microencapsulated Forms in Intestinal Diseases—Current Clinical Approach

**DOI:** 10.1007/s10620-025-09536-4

**Published:** 2025-11-14

**Authors:** Miłosz Caban, Ewa Pikus, Karolina Czarnecka-Chrebelska, Katarzyna Oszajca, Monika Witusik-Perkowska, Jagoda Dudek, Janusz Szemraj, Ewa Brzeziańska-Lasota, Renata Talar-Wojnarowska

**Affiliations:** 1 Department of Digestive Tract Diseases, Medical University of Lodz, Kopcińskiego 22, 90-153 Lodz, Poland; 2 Department of Biomedicine and Genetics, Medical University of Lodz, Lodz, Poland; 3 Department of Medical Biochemistry, Medical University of Lodz, Lodz, Poland

**Keywords:** Diverticular disease, Inflammatory bowel diseases, Irritable bowel syndrome, Microencapsulated SB, MSB, Postbiotic

## Abstract

**Background:**

In recent years, the importance of butyrate in prevention and health promotion in human health has been revealed and many publications have highlighted its role as a key component for intestinal functioning. Recent findings show that sodium butyrate has anti-inflammatory and immuno-modulatory activities in intestinal diseases and may be used in the therapy of intestinal diseases. Sodium butyrate mitigates mucosal inflammation and oxidative status, restores the damaged epithelial barrier, and modulates visceral sensitivity and intestinal motility. Novel forms of encapsulation are being developed to improve the effectiveness of sodium butyrate, as well as its palatability and patient’s compliance

**Methods:**

We conducted a comprehensive literature review. In this review, we discuss the utility, efficacy and safety of sodium butyrate preparations, including different microencapsulated forms in the management of main intestinal diseases, primarily inflammatory bowel diseases, irritable bowel syndrome and diverticular disease. .

**Results and Conclusions:**

Advanced microencapsulated sodium butyrate preparations seem to be an promising form that could be used as add-on therapy for intestinal diseases. Due to butyrate’s rapid epithelial absorption and local activity in the digestive tract, clinical outcomes may depend not only on the active ingredient but also on the delivery technology, release profile, and dosage. Therefore, we suggest that clinical results should be assessed in relation to specific preparations. Our summary confirms that specific microencapsulated versions, including those utilizing the MSB^®^ technology are valuable therapeutic options supporting the treatment of intestinal diseases. Differences between clinical study results suggests that formulation of butyrate impacts its efficacy.

## Introduction

Nowadays, the application of short-chain fatty acids (SCFAs) in gastroenterology is growing in popularity, which may be the result of the desire to improve the effectiveness of therapy for individual diseases. SCFAs are microbial metabolites that are formed as products of the fermentation of dietary fiber by intestinal anaerobic bacteria. Acetate (C2), propionate (C3) and butyrate (C4) are the main SCFAs and are produced in the colonic lumen in different proportions with an appropriate ratio of 3:1:1, respectively [1]. Their concentrations in the intestinal lumen depend on fiber intake, the composition of gut microbiota and its capacity for fermentation, intestinal absorption, and transit time [2]. Of these three, butyrate is a key bacterial metabolite and is considered the most important for health. Butyrate provides multiple pro-health effects on the human body and has a potential therapeutic effect against various intestinal diseases, mainly anti-inflammatory. Butyrate can be administered as a postbiotic—a preparation of non-living microorganisms and/or their metabolites or cell components that have health-promoting effects confirmed in clinical trials. Specifically, butyrate is classified as a metabolic postbiotic containing only metabolites or end products of microorganisms without cell fragments such as cell membrane lipids, cell wall compounds, or surface structures [3]. Currently, sodium butyrate (SB), the commonly applied form of butyrate, is increasingly used as a supportive agent in the treatment and prevention of various diseases and disorders of the digestive tract, mainly inflammatory diseases, specific or non-specific diarrhea, functional disorders, dysbiosis-related disorders, and post-surgery, post-chemotherapy or post-radiotherapy conditions [4, 5]. However, the SB efficacy is limited by some factors impairing its palatability and bioaccessibility. The unpleasant taste and odor of rancid butter of SB dictate the necessity of developing protected forms which improve tolerance and patient compliance. Due to the local activity of butyrate and its quick absorption by the epithelium if the digestive tract, the beneficial effects of SB on both small and large intestine, it is necessary to ensure a gradual and sustained release along the entire intestine to achieve optimal effects. That is why novel forms of SB are still being developed. Noteworthy is microencapsulated SB and patented microencapsulated form of SB (MSB^®^), which effectiveness and tolerance are satisfactory. Studies suggest that MSB^®^ supplementation may be applied as an additional treatment with high efficacy improving the results of intestinal diseases therapies [6–12].

Hence, this review discusses the utility of SB, MSB^®^ and other microencapsulated or otherwise protected forms of SB in gastroenterological diseases and briefly summarizes actual knowledge about the use of SB in intestinal disease. Also, we would like to discuss the technological challenges associated with its oral administration, as well as assess whether the use of SB is effective in achieving for obtaining clinical improvement in major intestinal diseases.

## Search Strategy

PubMed, Google Scholar, Wiley, Springer, Scopus, Embase, and Web of Science databases were systematically and extensively searched for the bibliography. Clinical trials were also searched using the ClinicalTrials.gov database. The search included all the studies published up to August 2025, using the following keywords, alone or in combination: microencapsulated sodium butyrate, sodium butyrate, MSB, encapsulation, bowel, bowel diseases, gut diseases, intestinal diseases, inflammatory bowel disease, Crohn’s disease, ulcerative colitis, irritable bowel syndrome, diverticular disease, small intestinal bacterial overgrowth, colitis, radiotherapy-induced colitis, chemotherapy-induced colitis, diarrhea, constipation, effectiveness, safety, adverse effects. Articles concerning SB, microencapsulated SB and MSB^®^ were included. Articles concerning other forms of SB protection than microencapsulation and animal studies were excluded. The searches were filtered to include only studies published in the English language. The titles and abstracts were independently screened by the investigators, and the selected papers were subsequently discussed with all the authors. Such a selection process ensures that this review is based on relevant research. All-randomized studies were included in this article. We thoroughly evaluated each article, assessing the methodologies, results, and implications, which enabled us to summarize the current knowledge in the field, identify gaps, and propose directions for future research.

## Health-Promoting Properties of SB and Rationale for Its Use in Intestinal Diseases

Butyrate serves as an important cell signaling molecule that modulates numerous physiological pathways important for maintaining intestine health. Also, it exerts a lot of pro-health activities supporting intestinal homeostasis. SB enhances the intestinal epithelial barrier by upregulation of the expression of tight junction proteins, and in the case of damaged intestinal epithelial barrier, butyrate stimulates restoration, the regeneration and healing of damaged intestinal epithelium [13, 14]. In consequence, SB maintains a physiological mucus layer and adequate permeability of the intestinal barrier [13, 14]. Furthermore, SB reinforces the intestinal defense barrier by stimulating production of mucins and antimicrobial peptides, such as defensins and cathelicidin (LL-37) [13, 15]. SB possesses anti-inflammatory activity by stimulation of anti-inflammatory cytokines production and inhibition of nuclear factor kappa B (NF-κB) signaling pathway, a key pathway promoting intestinal inflammation and expression of pro-inflammatory cytokines and enzymes. SB activates free fatty acid receptor 2 (FFAR2) and free fatty acid receptor-3 (FFAR3) in the colon and small intestine, leading to production of anti-inflammatory cytokines, including IL-18 [15]. The activity of SB is complex, and the anti-inflammatory effect is accompanied by a strong anti-oxidative effect. SB has been shown to reduce DNA damage from oxidative stress in both human and rat-derived colonocyte cultures [16]. Also, SB was able to modulate pro-inflammatory signals and inhibit several nucleotide-binding oligomerization domain-like receptor-3 (NLRP3) inflammasome markers in an in vitro co-culture model of intestinal inflammation [16]. SB upregulates the production of transforming growth factor-beta 1 (TGF-β1), a cytokine promoting anti-inflammatory regulatory T cells (Treg), in intestinal epithelial cells [17]. Moreover, the mitigating oxidative stress by SB may be mediated by the inhibition of production of nitric oxide (NO), a neurotransmitter and immunomodulator. SB decreased NO synthesis in neutrophils isolated from rats and cultured in vitro [18]. It was proven that SB exhibits anti-cancer activity. SB promoted the apoptosis of HCT116 cells, colorectal cancer (CRC) cells, through activating the caspase-cascade, a critical pathway for programmed cell death [19], as well as exerted in vitro anti-proliferative effect against CRC cells through inducing cell cycle arrest at the G2 phase with a drop in S-phase fraction (including c-Myc/p21 signaling) [20]. In contrast, SB stimulates proliferation, differentiation and maturation of healthy enterocytes and colonocytes, as well as enhances growth of intestinal villi and causes an increase in crypt depth. Moreover, SB exerts a trophic effect on the ileal and jejunal epithelial cells, probably indirectly through a neurohormonal mechanism [13, 14, 18]. On the other hand, butyrate is the main energy substrate for enterocytes and colonocytes, where it is metabolized in the mitochondria by β-oxidation to produce NADH, H + and acetyl-CoA, consequently further used to generate ATP in the Krebs cycle [21]. In addition, SB reduces the risk of gut microbiota disturbances and contributes to diversity and abundance of intestinal microbiota. It promotes the growth of commensal SCFA-producing bacteria, such as *Faecalibacterium prausnitzii* and *Roseburia* spp., and prevents colonization of pathogenic bacteria, such as *Escherichia coli* and *Clostridioides difficile,* by decreasing the pH value of the intestinal tract [22]. Moreover, SB improves gut motility, disturbed in the most of inflammatory intestinal diseases, through neuroendocrine activity, maintenance of normal intestinal smooth muscle tone and visceral sensitivity of sensory receptors [13, 14]. The above data indicate that SB has pleiotropic activity. It contributes to the proper functioning of the intestine and enables the proper development of intestinal microflora. It seems that probiotics may require butyrate for adequate microbial colonization in intestinal diseases. In the case of dysbiosis, an inappropriate composition of intestinal microbiota observed in some intestinal diseases, there is a decrease in butyrate production, leading to further intensification of disorders of intestinal microbiota, intestinal barrier and intestine function. Butyrate can enable the recolonization of bacteria by the proper microbiota, acting synergistically with probiotics [18]. In view of the above data about disorders observed in the described intestinal diseases and mechanisms of butyrate action, probiotics and prebiotics are not able to provide the supplementation of butyrate, especially in the initial stage of restoring balance in the intestines affected by dysbiosis and inflammation. Moreover, the use of butyrate may be needed to achieve adequate therapeutic effect during probiotic and prebiotic therapy. Therefore, it may be necessary to use simultaneously probiotics and postbiotics—in the form of SB.

## SB Supplementation: Administration Route, Clinical Relevance, Limitations and Microencapsulated Forms

As we have mentioned before, butyrate is produced from fiber during bacterial fermentation by butyrogenic bacteria such as *Roseburia* spp., *Blautia* spp., *Faecalibacterium prausnitzii*. In intestinal diseases and disorders lower abundance of butyrogenic bacteria and lower butyrate level is observed. Therefore, the oral intake of a high amount of fiber may not be a sufficient source of butyrate. It is essential to note that, despite its beneficial health properties, a high-fiber diet may cause gastrointestinal discomfort such as gas, bloating and constipation. Also, consumption of foods or supplements rich in dietary fiber has many health benefits, but is not without drawbacks such as quality fluctuations, short shelf life, heterogeneous effects and low compatibility to immunocompromised people. That is why butyrate supplementation appears to be a suitable alternative. Butyrate supplements are safe and stable, easy to store and are associated with a lower risk of antimicrobial resistance [23, 24]. Various forms of butyrate, such as SB, calcium butyrate, lysine butyrate, tributyrin or crystalline butyrate, as well as various pharmaceutical forms of preparations, such as capsules, microcapsules, sachets, rectal enemas, suppositories are available [4, 25, 26]. Pure butyrate may not be used because of its unpleasant taste, odor of rancid butter, and instability. Calcium butyrate and derivatives like 4-phenylbutyrate, although less studied, offer different solubility and absorption profiles [16]. In consequence, SB in capsules or sachets is the most commonly used in practice due to its good solubility in water and milder odor. The rectal suppositories are the second most prevalent form of SB administration and are used primarily in ulcerative colitis (UC) and radiotherapy-induced proctitis. It is worth emphasizing that the extent of rectal enemas activity is limited to terminal parts of the large intestine. In addition, the rectal administration of SB is less preferred by patients compared to oral application. Also, studies indicate that rectal infusions may disrupt the gut microbiota due to the carrier of SB [27]. That is why oral administration of SB is dominant in gastroenterological disorders.

The effective delivery of oral SB to the site of demand and its absorption is relatively problematic and largely depends on its formulation. It is worth emphasizing that the oral administration of preparations in some formulations containing butyric acid salts does not cause the delivery of an appropriate amount of the substance to the colon due to fact that butyric anion is rapidly absorbed in the stomach and initial parts of the small intestine [28]. Ensuring that the butyrate reaches both the small and large intestines is critical for its therapeutic efficacy. In addition, SB dissociates rapidly, and the butyrate anion is absorbed almost immediately after release. Moreover, adequate formulation of SB affects taste and odor, which significantly determine patient adherence to supplementation [10, 29, 30]. On the other hand, digestive enzymes, as well as disturbances in gut motility, gut microbiota composition and digestive juices pH, observed in patients with intestinal diseases, especially inflammatory diseases, impair different SB formulations release and reduce their bioaccessibility [31]. In order to avoid this undesirable effect, SB may be effectively delivered through microencapsulation technology involving the use of additional cross-linking substances, facilitating the controlled release of SB in different sections of the digestive tract, with the predominant amount being released in the distal part of small and large intestine [23, 24]. Individual preparations differ in the efficacy of butyrate protection from premature absorption in the gastrointestinal tract. The choice of formulation influences its release pattern, patient tolerance, and effectiveness in clinical studies [32].

Several studies have investigated the efficacy of different SB formulations, highlighting significant variations in their release profiles, absorption rates, and clinical impact. Noteworthy is MSB^®^, which is characterized by uniform, gradual release profile at different parts of the digestive tract. In addition, it contains a complex lipid matrix structure, which leaves this formulation with the sustainable release profile of SB throughout the small and large intestine. That is why MSB^®^ seems to be one of the most efficient SB encapsulations for achieving clinical effects in intestinal diseases.

## Potential Use of SB and Microencapsulated Forms in Intestinal Diseases—Clinical Studies

As mentioned above, SB has a lot of pro-health activities that could be used in the therapy of some intestinal diseases associated with inflammatory response, disorders of the intestinal barrier, and disturbances in gut microbiota composition. It seems that the use of SB in irritable bowel syndrome (IBS), inflammatory bowel diseases (IBD), especially UC, and diverticular disease (DD) may be beneficial, mainly by mitigating symptoms. In radiotherapy-induced colitis, small intestinal bacterial overgrowth (SIBO) or diarrhea associated with antibiotics, clinical trials aimed to the utility of SB are still in progress; however, actually there is no strong evidence.

The literature review revealed a number of studies in the context of the utility and application of butyrate in intestinal diseases, mainly IBS and IBD [6, 32]. IBS is one of the most commonly diagnosed chronic functional gastrointestinal diseases. The presence of abdominal pain, bloating, constipation and/or diarrhea without any morphological and biochemical changes is typical [33]. Data show that disturbances of gut microbiota and intestinal barrier may take part in the pathogenesis of IBS, suggesting that the use of butyrate may be helpful. There are several studies presenting variable results about the significance of gut microbiota in IBS [34]. Nonetheless, colonization of the non-resident bacteria and an overgrowth of the resident bacteria in the small intestine may be associated with the symptoms of IBS. Generally, the composition of gut microbiota is dependent on the type of IBS. In the IBS dominant with diarrhea (IBS-D), significant reduction in abundance of *Actinobacteria* and *Bifidobacterium,* as well as increased abundance of *Lactobacillus*, *Streptococcus*, *Pediococcus*, *Enterobacteriaceae*, including Escherichia coli, were observed compared to the healthy subjects [34]. In contrast, patients with IBS dominant with constipation (IBS-C) were characterized by elevated amount of *Enterobacteriaceae*, *Veilonella*, sulfate-reducing bacteria concomitant with lowered levels of *Eubacteria*, *Bifidobacterium* and *Lactobacillus* in comparison to healthy control subjects [35]. These changes in gut microbiota impair the mucosal immune system [36]. On the other hand, there is evidence demonstrating the presence of intestinal barrier dysfunction in IBS determining the clinical course of the disease. The increased intestinal and colonic permeability contributing to immune activation was documented in the subjects with IBS. In addition, the loss of intestinal barrier function positively correlated with the intensification of abdominal pain and diarrhea [37]. The changes described above may justify the use of butyrate in IBS. However, data focused on the microbial butyrate production efficiency profile in patients with IBS is not clear. Nevertheless, restrictive dietary interventions, such as the low-FODMAP diet, limit the supply of readily fermentable oligo-, di-, monosaccharides, and polyols, which are key substrates for bacterial fermentation in the colon. It contributes to the improvement of IBS symptoms. Reducing the amount of available indigestible carbohydrates reduces the rate and intensity of fermentation, resulting in reduced gas production and intra-intestinal pressure, thereby alleviating bloating, abdominal pain, and discomfort [38, 39]. Simultaneously, limiting the availability of substrates for bacteria of the genera *Faecalibacterium* and *Roseburia* and other key butyrate producers leads to a decrease in the concentration of SCFA, including butyrate, in the intestinal lumen [40]. SB supplementation or interventions increasing the relative abundance of butyrogenic bacteria may compensate for the decrease in its endogenous production in patients following a low-FODMAP diet, which promotes improved intestinal barrier integrity, reduced inflammation, and further alleviated clinical symptoms [10, 41].

In the context of IBS, some clinical trials using MSB^®^ and other microencapsulated SB forms were performed. The randomized, placebo-controlled clinical trial analyzed whether oral supplementation with MSB^®^ as an additive therapy in patients with IBS could be efficient for mitigating the disease symptoms. Researchers hypothesized that the additive therapy enriched with MBS^®^ may contribute to the improvement of the therapeutic effectiveness of standard IBS treatment with mebeverine, simethicone, or trimebutine depending on the disease form. The subjects consumed one capsule containing 150 mg MSB^®^ two times per day. The six-week administration with MSB^®^ resulted in a significant decrease of abdominal pain and discomfort severity, defecation disorders, as well as improvement of the quality of life, measured using IBS-QoL questionnaire, in comparison to the group treated without MSB^®^ (placebo group) (*p* < 0.0001) [6]. The next clinical trial also assessed the use of MSB^®^ as an addictive therapy of IBS. The human study included a population of 66 adult subjects with diagnosed IBS who were administered mebeverine, trimebutine or lubricating and swelling agents that facilitate the passage of stools. Results demonstrated a significant clinical improvement in the context of presence and severity of clinical symptoms, evaluated using the Birmingham scale, after twelve-week therapy compared to the standard therapy without MSB^®^. Interestingly, the authors of this trial evaluated the effectiveness of the therapy with MSB^®^ using also a single question (*“Did you have adequate relief of abdominal pain or discomfort in the past week?”*). The results based on answers revealed significantly better outcomes of the therapy in the MSB arm compared to the placebo arm after one month (*p* = 0.0084) and three months of treatment (*p* = 0.0087), and even two months after the end of treatment (*p* = 0.0023). However, these results were not accompanied by the improvement of the quality of patients’ life [7]. The same research group conducted on IBS patients in Poland analyzed the association between the administration of MSB^®^ and the presence and mitigation of pain in IBS. The team performed assessment after four and twelve weeks of therapy, whereas the therapeutic intervention duration was twelve weeks. After four weeks of study, a significant reduction in abdominal pain during defecation was observed in the MSB^®^ arm in comparison to the placebo arm. It is worth emphasizing that greater beneficial effects were noted after twelve weeks of therapy. After this time, the MSB^®^ group was characterized by statistically significant reduction in the frequency of occurring spontaneous abdominal pain (*p* = 0.0132), postprandial abdominal pain (*p* = 0.031) and abdominal pain during defecation (*p* = 0.0002) as opposed to the placebo group. In addition, the significant decrease in the occurrence of constipation (*p* = 0.0493) and urgent sensation after defecation (*p* = 0.0417) was shown after twelve-week treatment with MBS^®^. Furthermore, a subjective assay of changes of abdominal pain based on a single closed-end question (yes or no answer) *“Did you observe adequate relief of IBS symptoms related to abdominal pain or discomfort within the past week?”* was performed. After both four (32% *vs.* 6.25%, *p* < 0.01) and twelve weeks (53% *vs.* 15.6%, *p* < 0.01), the greater number of patients with IBS in the MSB^®^ group reported relief in IBS symptoms compared to the placebo group [8]. In the year 2022, Lewandowski et al. determined the effect of MSB^®^ supplementation on reducing the severity of clinical symptoms and improving quality of life in subjects with IBS. It was performed as a multicenter cross-sectional study to evaluate the effects of capsules containing 150 mg of MSB^®^, administered twice a day for twelve weeks. The intensity of self-reported gastrointestinal symptoms, the frequency of abdominal pain, diarrhea and constipation, and previous treatment were analyzed in the group of 3000 patients, among whom the IBS form with variable bowel movements was dominant (39.29%), followed by diarrhea form (29.54%), constipation form (21.93%) and unclassified (9.24%). The MSB^®^ treatment resulted in significantly reduced severity of abdominal pain related to defecation, a change in the bowel movements frequency, a change in the stool consistency, as well as postprandial pain, and spontaneous pain (*p* < 0.001). Additionally, the twelve-week intake of MSB^®^ significantly diminished the occurrence of flatulence, diarrhea, constipation, urgent pressure for bowel movements, nausea and vomiting (*p* < 0.001). Moreover, the authors also determined that the treatment with MBS^®^ was better tolerated (*p* < 0.001), more effective (7.4 *vs.* 3.39; *p* < 0.001), less expansive (7.78 *vs.* 4.81; *p* < 0.001) than previous used IBS therapy (*p* < 0.001). Also, MBS^®^ improved quality of the life (3.66–6.61, *p* < 0.001), social life (3.73–6.43, *p* < 0.001), professional work (3.41–6.08, *p* < 0.001), life comfort (3.31–5.8, *p* < 0.001), and these data were measured using adequate questionnaires with a scale from 0 to 10 points [10].

IBD, including UC and Crohn’s disease (CD), are important representatives of gastrointestinal diseases. In their course, alterations of gut microbiota, as well as reduced level of SCFA, including butyrate, are observed [42]. It was proven that exacerbation of UC and its active form are associated with significantly lowered butyrate levels, while patients in remission are characterized by increased levels of butyrate [42]. Moreover, dysbiosis is known as one of the risk factors of IBD occurring. The most significant changes in the microbiota composition of IBD subjects are the reduced diversity in bacteria species related to diminished abundance of *Bacteroidetes* and *Firmicutes* concomitant with rise in the abundance of *Proteobacteria* or *Escherichia coli* [43]. Also, a decreased number of butyrate-producing bacteria, such as *Roseburia* spp. or *Faecalibacterium prausnitzii*, occur in the course of IBD [44]. It demonstrates the potential utility of butyrate administration in IBD. Enemas with butyrate were used in UC and were demonstrated to reduce stool frequency or disease activity index [45–47]. Interestingly, a most recent systematic review of randomized controlled trials performed in year 2021 proved that enemas with SB did not significantly contribute to the improvement of endoscopic and histological scores in UC, showing that they do not seem to be support in the therapy of UC. In the context of CD, there are no reliable data according to the effectiveness of butyrate enemas [48]. Also, in the year 2022, one randomized placebo-controlled multicenter trial aimed at the assessment of efficacy of oral butyrate in IBD was performed. In this study, a 12-week administration using microencapsulated SB, as adjunctive therapy, did not demonstrate effectiveness in newly diagnosed children and adolescents with IBD [32].

On the other hand, in 2025, Goldiş et al. assessed clinical efficacy of MSB^®^ as an adjunct to conventional treatment in children and adolescents with UC and CD aged 7–18 years. The 12-week oral supplementation of 150 mg MSB^®^ contributed to significant clinical improvement. A greater number of patients experienced remission by week 12 of the study in the treatment group compared to the placebo group (47.73% in placebo group vs 81,82% in study group). At 12 weeks, the MSB^®^ group had the significantly lower CRP levels (18.14 ± 11.19 mg/L) compared to the placebo group (57.00 ± 33.28 mg/L) (*p* < 0.001). Fecal calprotectin (FCP) decreased much more after 12 weeks in the treatment group than the placebo (*p* < 0.001), suggesting that the MSB^®^ group was better able to regulate intestinal inflammation. During the study, no adverse effects were reported by any of the patients [12]. For comparison, a similar study conducted on the basis of a similar research protocol in a similar study group did not show a positive effect of supplementation [32]. Therefore, the results of the study suggest that MSB^®^ has an advantage over other microencapsulated formulas, and MSB^®^ may be used as supplement add-on to conventional therapy of IBD facilitating achievement of clinical remission.

So far, there are few more evidence about the utility of microencapsulated SB in IBD. It was proven that the oral microencapsulated SB supplementation contributed to an increase of the growth of SCFA-producing bacteria (*Lachnospiraceae* spp.) in patients with UC, as well as the butyrogenic colonic bacteria (*Butyricicoccus*) in subjects with CD, improving the quality of life [29]. Moreover, the microencapsulated SB supplementation additional to mesalamine increased the probability of maintaining remission of UC, defined as Mayo partial score ≤ 2 and fecal calprotectin (FCP) < 250 µg/g at 12 months of follow up, compared to therapy with only mesalamine, indicating on high efficacy of microencapsulated SB add-on therapy [49].

Diverticular disease (DD) is globally common disease, and its prevalence of colonic diverticula is about 5% before the age of 40. However, its frequency increases as we age while to be above 70% after 80 years. Data show that the low-grade inflammation, immune activation and disturbances in gut microbiota may have some role in the etiopathogenesis and development of DD [50]. Initial pilot studies revealed that some bacterial species may be changed in symptomatic uncomplicated diverticular disease (SUDD), DD or diverticulosis. Pilot study performed by Barbara and co-researchers showed that the patients with SUDD were characterized by a significant decrease of fecal *Clostridium cluster IX*, *Fusobacterium* and *Lactobacillaceae* species in comparison to the subjects with diverticulosis [51]. Also, the high intensity and severity of bloating in the subjects with SUDD were associated with reduced fecal amount of *Roseburia*, producing butyrate that enhances intestinal motility and reduces hypersensitivity [52]. Nevertheless, the most recent systematic review demonstrated that there is no convincing confirmation of microbial dysbiosis in diverticulosis, DD and SUDD [53], which was validated in a population-based colonoscopy study [54].

Two-center, parallel, double blinded, randomized, placebo-controlled, per-protocol clinical trial was performed to assess MSB^®^ in seventy-three patients with diverticulosis confirmed by colonoscopy or barium enema or CT colonography. MSB^®^ was administered at dose of 150 mg twice a day for twelve months. This therapy contributed to significantly reduced number of diverticulitis episodes (6.67% *vs.* 31.8%, *p* < 0.05) in comparison to the placebo group, leading to the reduction in the number of performed abdominal imaging, mainly ultrasound examinations. Also, it was responsible for the improvement of the quality of patients’ life, expressed mainly by relief in abdominal pain and discomfort (55.67% *vs.* 22.73%, *p* = 0.0143) compared to the placebo group, whose participants received identical capsules without MSB^®^. Importantly, this intervention was not associated with adverse effects [55]. In addition, the literature review revealed a retrospective study by Tursi et al. (2025). The three-month oral application of microencapsulated and colonic-release formulation of SB led to a relevant increase in the diversity of gut microbiota in the patients with SUDD. The treatment contributed to the increase in SCFA-producing bacteria, as well as in taxa with dubious pathogenic potential. However, these gut microbiota changes were associated with significant reduction of abdominal pain improving quality of the patients’ life [56].

Notable disorders for which butyrate could potentially be used are radiotherapy- and chemotherapy-induced colitis. Radiotherapy and chemotherapy used due to cancer diseases may cause intestinal mucositis, as well as significant disturbances in the composition of gut microbiota, which in turn exacerbate inflammatory response of the mucosa. These changes contribute to oxidative stress, increased intestinal permeability, changes in mucus production and impairment of epithelial repair and immune response. It is known that the presence of SCFA-producing bacteria, as well as butyrate, facilitates intestinal homeostasis and mucosal healing [57]. Also, in chemotherapy-induced colitis, direct application of SCFA, including butyrate, has been demonstrated to elevate the level of anti-inflammatory IL-10 and reduce the production of pro-inflammatory IL-12 and TNF-α in the intestine. In addition, it was observed that butyrate with chemotherapeutics may promote anti-cancer immunity, prevent the development of chemotherapy-induced colitis, and improve efficacy of these drugs and extend progression-free survival during chemotherapy [58, 59]. On the other hand, the utility of sodium butyrate was analyzed in radiation-induced proctitis. In 2013, Stojcev et al. proved that enemas with butyrate are able to prevent radiation-induced proctitis from occurring during radiotherapy due to prostate or cervical cancer. Also, in the same study, the therapeutic effect of SB enemas in radiation-induced proctitis was demonstrated by the reduction of reported rectal bleeding and the necessity of therapy intensification [60]. In contrast, two other clinical trials did not confirm beneficial outcomes of SB enemas for radiation-induced proctitis, showing the lack of effects on rectal bleeding, severity of the disease, rectal pain, or endoscopic and histological improvement [61, 62]. So far, there is one study assessing the use of microencapsulated SB in the context of radiotherapy-induced proctitis. Cannizzaro et al. (2025) evaluated the efficacy of microencapsulated SB in the prevention of acute radiotherapy-induced proctitis in men treated with radiotherapy due to prostate cancer. The oral administration of microencapsulated SB began one week before and ended four weeks after the end of radiotherapy. The results indicated that microencapsulated SB appeared effective in reducing radiotherapy-induced bowel toxicity, minimizing stool changes, incontinence, and abdominal pain. Although patients’ health perception declined at radiotherapy completion, it improved after one month, suggesting microencapsulated SB may support clinical recovery post-treatment [63].

The literature review also revealed one interesting study in the context of the MSB^®^ use. A prospective, randomized, placebo-controlled double-blind study performed in 2024 year analyzed the impact of MSB^®^ on diabetes mellitus (DM) control, reduction of gastrointestinal dysfunction manifestation and SIBO occurrence in patients with type 2 DM treated with diet or oral hypoglycemic drugs. It is worth emphasizing that gastrointestinal symptoms and SIBO are more common in the subjects with DM. After the oral intervention with MSB at the dose of 1500 mg per day, the relevant relief in abdominal pain (*p* = 0.001), as well as significant reduction of diarrhea (*p* = 0.001), constipation (*p* = 0.012), flatulence (*p* < 0.001) and decrease of SIBO prevalence (*p* = 0.001) were observed in the comparison to the placebo group. Interestingly, MSB^®^ also contributed to better DM control, the therapy was associated with significant improvement of body mass index (*p* < 0.001) and HbA1C level (*p* = 0.002) [11]. The above presented data clearly indicated the utility and effectiveness of MSB^®^ in intestinal diseases, mainly IBS. Nevertheless, large-scale well-controlled human clinical trials are necessary to establish the health-promoting effects of consuming MSB. On the contrary, Bouter et al. in 2018 conducted study in lean and metabolic syndrome male patients with oral supplementation of SB (4 g per day for 4 weeks). This clinical study, even though using high amount of butyrate didn’t show positive effect in metabolic syndrome patients [64]. This lead to the conclusion that it is difficult to draw up recommendations for human populations based only on these findings and product-specific release profile should be taken into consideration in further clinical studies.

**Fig. 1:**
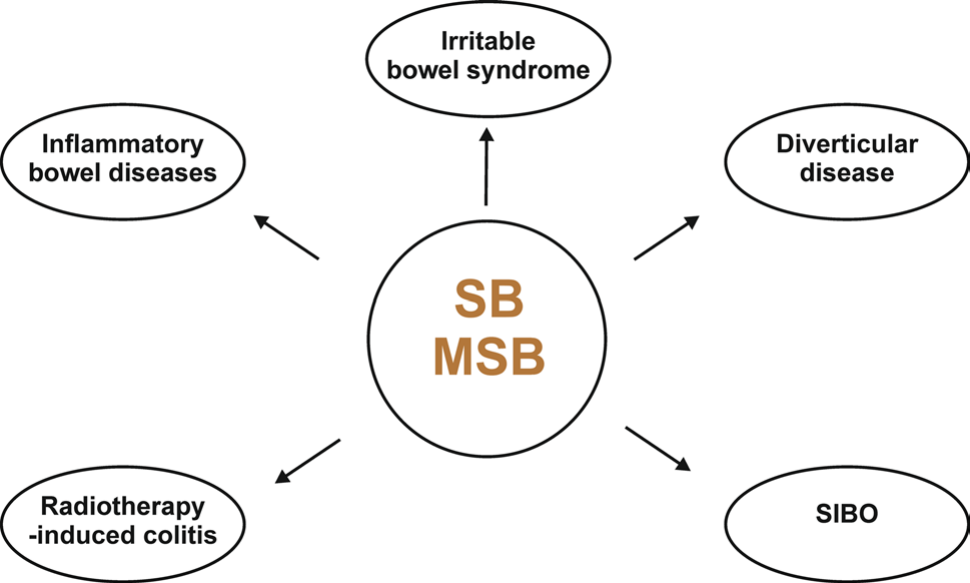
A schematic view of intestinal diseases, whose treatment could be supported by the MSB supplementation. *SB* sodium butyrate

The most presented studies analyzed use of SB, no microencapsulated SB or MSB. There is insufficient and limited data confirming the effectiveness of MSB^®^ in intestinal disease. The available studies suggest that MSB^®^ formulation may be promising for its use in some intestinal diseases. Selected studies investigating the effectiveness of SB and microencapsulated SB/MSB in intestinal diseases are summarized in Tables 1 and 2, respectively. In addition, we summarized the potential intestinal diseases that could be targets for the use of SB in the treatment in Fig. 1.

**Table 1:** Overview of clinical studies related to non-microencapsulated butyrate

Disease	ClinicalTrials.gov identifier /phase (if specified)	Participants/enrollment	Dosage / duration	Clinical effects/findings	References
UC	Single blind with crossover	Ten patients with UC	100 mL, 100 m sodium butyrate, 40 mM NaCl, pH 7.0, twice a day for 2 weeks	↓ stool frequency ↓ endoscopic score ↓ inflammation histopathological degree ↓ disease activity	[46]
UC	Open label study	Ten patients with distal UC	60 mL, 80 mM sodium butyrate, pH 7.0, daily for 6 weeks	↓ disease activity	[45]
UC	Randomized clinical trial	Thirty-eight patients with left-side UC; treatment group (*n* = 19) or placebo group (*n* = 19)	60 mL, 80 mM sodium butyrate, pH 7.0, daily for 6 weeks	↔ clinical remission	[65]
UC	Randomized clinical trial	Forty-seven patients with active distal UC	60 mL, 100 mM sodium butyrate, pH 5.5, twice a day for 4–8 weeks	↔ disease activity	[66]
Radiation-induced proctitis	Randomized, double-blind, placebo-controlled, cross-over pilot trial	Fifteen patients with radiation chronic proctitis	40 mM sodium butyrate, twice a day for 2 weeks	↔ rectal pain, rectal bleeding, diarrhea ↔ endoscopic score ↔ histological score	[61]
UC	Randomized clinical trial	Eleven patients with distal UC; treatment group (*n* = 6) or placebo (*n* = 5)	60 mL, 100 mM sodium butyrate, pH 5.5, twice a day for 8 weeks	↓ disease activity index ↓ NF-κB in lamina propria macrophages	[47]
Radiation-induced proctitis	Randomized prospective placebo controlled trial	One hundred patients (I group—fifty patients in prevention group and II group—fifty patients in treatment group)	I group—150 mL enema with sodium butyrate once a week for 3 months, started 2–14 days after radiation due to the prostate or cervical cancer II group—150 mL enema with sodium butyrate once a week for 3 months after diagnosis	I group: ↓ radiation-induced proctitis occurrence II grouop: ↓ rectal bleeding, clinical symptoms, therapy intensification	[60]
Radiation-induced proctitis	Multicenter randomized placebo-controlled dose-finding phase 2 study	One hundred sixty-six patients with prostate cancer during radiotherapy	Rectal enema with 1, 2 or 4 g of sodium butyrate daily during and two weeks after radiotherapy	↔ incidence, severity and duration of acute radiation proctitis	[62]

↑ activation or increase, ↓ inhibition or decrease, ↔ no impact, *CD* Crohn’s disease, *IBD* inflammatory bowel disease, *IBS* irritable bowel syndrome, *NF-κB* nuclear factor kappa B, *UC* ulcerative colitis

**Table 2:** Overview of clinical studies related to microencapsulated SB and MSB

Disease	ClinicalTrials.gov identifier /phase (if specified)	Participants/enrollment	Dosage / duration	Clinical effects/findings	References
IBS	Randomized, placebo-controlled clinical trial	Fifty-nine patients with IBS; treatment group (*n* = 30) or placebo group (*n* = 29)	MSB^®^; 300 mg per day (2 × 150 mg) orally for 6 weeks	↓ abdominal pain and discomfort ↓ defecation disorders ↑ quality of the life ↔ bloating severity	[6]
IBS	Two-center, randomized, placebo-controlled clinical trial	Sixty-six patients with IBS; treatment group (*n* = 34) or placebo group (*n* = 32)	MSB^®^; 300 mg per day (2 × 150 mg) orally for 12 weeks	↓ symptoms ↔ quality of the life	[7]
IBS	Parallel, double-blinded, randomized, placebo-controlled per-protocol clinical trial	Seventy-nine patients with IBS; treatment group (*n* = 41) or placebo group (*n* = 38)	MSB^®^; 300 mg per day (2 × 150 mg) orally for 12 weeks	↓ abdominal pain ↓ constipation	[8]
Diverticulosis	Two-center, parallel, double-blinded, randomized, placebo-controlled, per-protocol clinical trial	Seventy-three patients with diverticulosis; treatment group (*n* = 37) or placebo group (*n* = 36)	MSB^®^; 300 mg per day (2 × 150 mg) orally for 12 months	↓ abdominal pain and discomfort ↓ diverticulitis ↑ quality of the life	[55]
IBD (CD and UC)	Double-blind, placebo-controlled, pilot study	Forty-nine patients with IBD (UC = 30; CD = 19)	Microencapsulated SB; 1800 mg per day (3 × 600 mg) orally for 60 days	↑ SCFA-producing bacteria (UC) ↑ butyrogenic colonic bacteria (CD) ↑ quality of the life (UC) ↔ FCP	[29]
UC	Observational, single-center, prospective cohort study	39 patients with UC (18 treated with MSB add-on therapy and 21 with standard mesalamine only)	Microencapsulated SB; 1000 mg per day (2 × 500 mg) orally for 12 months	↓ FCP ↓ partial Mayo score	[49]
IBS	Multi-center cross-sectional study	Three thousand patients with IBS	MSB^®^; 300 mg per day (2 × 150 mg) orally for 12 weeks	↓ abdominal pain ↓ diarrhea, constipation ↑ quality of the life	[10]
IBD (CD and UC)	Prospective, randomized, placebo-controlled multicenter study	Seventy-two patients with IBD (CD or UC); treatment group (*n* = 29) or placebo group (*n* = 43)	Microencapsulated SB; 150 mg sodium butyrate twice a day for 12 weeks	↔ disease activity ↔ remission rate	[32]
Diabetes mellitus/ SIBO	Prospective, randomized, placebo-controlled double-blind study	Fifty-two patients with type II diabetes mellitus; treatment group (*n* = 29) or placebo group (*n* = 23)	MSB^®^; 1500 mg per day (2 × 750 mg) orally for 12 weeks	↓ abdominal pain ↓ diarrhea, constipation ↓ SIBO	[11]
Diverticular disease	Retrospective study	Fifty-nine patients with symptomatic uncomplicated diverticular disease	Microencapsulated SB; Two capsules bid, for a total dose of 400 mg butyrate for 3 months	↑ gut microbiota diversity ↓ abdominal pain	[56]
IBD (CD and UC)	Single-center, prospective, randomized, placebo-controlled trial	Eighty-eight pediatric patients with CD and UC; treatment group (*n* = 44) or placebo group (*n* = 44)	MSB^®^; 150 mg per day orally for 12 weeks	↓ CRP level ↓ FCP ↓ disease activity	[12]
Acute radiotherapy-induced proctitis	Prospective single-center study	One hundred twenty-two men treated radiotherapy due to prostate cancer	Microencapsulated SB; Three capsules, for a total dose of 600 mg butyrate	↓ abdominal pain	[63]

↑ activation or increase, ↓ inhibition or decrease, ↔ no impact, *FCP* fecal calprotectin, *IBD* inflammatory bowel diseases, *IBS* irritable bowel syndrome, *SCFA* short-chain fatty acids, *SIBO* small intestinal bowel overgrowth

## Safety Profile of SB

Safety analyses provided some data about SB, including microencapsulated forms, such as MSB^®^ adverse effects; however, they are limited due to the small number of randomized clinical trials in which strict adverse events reporting is key. Nonetheless, the administration of different forms of SB seems to be very safe, also in elderly and pediatric population. There were no severe adverse events during SB and MSB® applications in all analyzed clinical trials. Transient mild nausea and diarrhea were the main reported adverse effects during SB supplementation, but they occurred in isolated cases [67]. Headache, abdominal distension, increase of blood pressure in hypertensive populations are other potential adverse effects reported during oral SB supplementation. These side effects usually cause that oral SB or microencapsulated SB supplementation is intermittent; however, it seems to be in less than 1% patients [67–69]. In the study of Lewandowski et al. treatment with MSB^®^ was also discontinued by some subjects due to the lack of a therapeutic effect. The rate of lack of efficacy may probably reach up 6%, but further research is needed [10].

## Summary and Conclusions

### Concluding Remarks

SB, microencapsulated SB, and especially MSB^®^ seem to be valuable options as supporting therapy for conventional treatment of selected intestinal diseases. Currently, published data confirm that microencapsulated SB and in particular MSB^®^ are effective, especially in IBS and in patients with IBD, DD or radiotherapy-induced colitis [6, 29, 55, 63]. It seems that SB, including MSB^®^, may be effectively applied as a support to conventional therapy and chance for achieving better clinical effects, but not the only form of treatment. Its application seems to be dedicated in subjects with stabile disease, mild or moderate manifestation of the disease or as prophylaxis of the recurrence of symptoms, but cannot be the basis of treatment. At the same time, it must be noted that different SB forms and presentations may have different release profiles and their dosages may vary. The encapsulated forms of SB, including MSB^®^, seem to be dedicated in diseases of large intestine, where inflammatory process is located in the distal part of the digestive tract, and the protection of SB against digestive processes is necessary [12, 55].

### Remaining Questions and Limitations

It is worth emphasizing that the discussed studies have some limitations. Different studies employed different dosages of SB, microencapsulated SB and MSB^®^, which may have contributed to variations in outcomes. There are no studies comparing doses of various forms of SB head-to-head. Based on available results, it seems that the use of high oral doses may not improve clinical effects, and it may associated with higher risk of side effects occurrence. In turn, different studies evaluating the efficacy of rectal SB employed various matrix of rectal forms, which may affect clinical findings. Also, other significant limitations of discussed studies are the relatively small size of the study groups and lack of multiinstitutional input. Currently, the number of studies in children is insufficient. Moreover, in the vast majority of studies, the assessment of effectiveness of SB and MSB^®^ was based on subjective clinical symptom improvement seemed practical, including self-reporting of symptoms on a questionnaire. Given that there are differences between, for example, the release of individual preparations, there is also a lack of studies directly comparing the effects of different preparations containing butyrate. Currently, the number of clinical studies, except those performed in IBS and IBD, is relatively small. Also, it cannot be clearly determined what form of SB is characterized by the greatest possible effectiveness and safety.

### Future Directions

Future directions for SB focus on optimizing its delivery, improving clinical trial design, and expanding its therapeutic applications to intestinal diseases. That is why further prospective, multicenter, randomized clinical trials are necessary to verify butyrate effectiveness in intestinal diseases. The long-term observation in clinical trials and determination of dosing regimens seem to be an important issue in the context of the next studies. In addition, there is a need to perform comparative studies with non-encapsulated SB head-to-head with microencapsulated SB, MSB^®^ and placebo to clarify efficacy of encapsulated forms of SB. Furthermore, assessment of the use efficacy of recommended therapeutic options compared to ones together with SB in individual intestinal disorders should be direction for future studies. Financial aspects and insurance issues may also have a relevant impact on the use of SB and its encapsulated forms. Intestinal diseases prevalence and consequently the use of healthcare resources is constantly increasing. However, there is no strong data about its financial burden in this indication. On the other hand, the data about safety and adverse effects is limited. That is why the detailed analysis of safety profile of SB supplementation should be determined. Future studies could consider to measure plasma or fecal levels at multiple time points to get more insight into the pharmacokinetics of oral SB and MSB^®^, as well as the effect of supplementation on the composition of the intestinal microbiota to determine molecular outcomes and adequate dose.

## Data Availability

Not applicable.

## Abbreviations

CD: Crohn’s disease
DD: Diverticular disease
BD: Inflammatory bowel diseases
IBS: Irritable bowel syndrome
MSB: Microencapsulated sodium butyrate
SB: Sodium butyrate
SCFA: Short-chain fatty acids
SIBO: Small intestinal bacterial overgrowth
UC: Ulcerative colitis
